# Advanced analysis of formulation parameters governing encapsulation efficiency: drug delivery system

**DOI:** 10.3389/fchem.2026.1794154

**Published:** 2026-07-07

**Authors:** Abdulrahman Sumayli, Saad S. Alqahtani

**Affiliations:** 1 Department of Mechanical Engineering, College of Engineering, Najran University, Najran, Saudi Arabia; 2 Sustainability Lab, Scientific and Engineering Research Center (SERC), Najran University, Najran, Saudi Arabia; 3 Clinical Pharmacy Department, College of Pharmacy, King Khalid University, Abha, Saudi Arabia

**Keywords:** causal inference, counterfactual analysis, encapsulation efficiency, intervention-oriented reasoning, niosomes, thin-film hydration

## Abstract

The encapsulation efficiency (%EE), used here as a consistently reported operational proxy for encapsulation performance in literature-derived datasets, in niosomal drug delivery systems has been analyzed using predictive modeling. However, these methods are hardly able to support intervention-oriented reasoning adequately. Therefore, the present study adopts a developed causal discovery framework on a data set of formulation from the literature to reveal assumption-aware causal structures connecting physicochemical drug properties, formulation composition, process conditions, and %EE. The input variables were selected based on consistent reporting across literature sources, physicochemical relevance to niosomal systems, and unambiguous availability in the extracted data, rather than outcome-driven criteria. From 116 observational samples of thin-film hydration only, causal graphs were generated through the combined use of constraint-based (PC Algorithm), score-based (Greedy Equivalence Search (GES)), and functional (Linear Non-Gaussian Acyclic Model (LiNGAM)) models. In common with the other methods, %EE was identified as a structurally downstream outcome. At the same time, the intrinsic drug properties (especially lipophilicity) were found to be upstream constraints rather than intervention levers. A notable systematic difference between the importance of features predictive of the model and the causal effect estimates was observed, underscoring that predictive relevance does not imply causal influence. Instead of dictating formulation rules, the framework allows for generating hypotheses, counterfactual exploration, and structured experimental planning under explicit causal assumptions.

## 1 Introduction

The objective of numerous scientific disciplines is to comprehend the mechanisms by which variables acquire their values (i.e., to establish a generative model) and to forecast those variables’ values when the mechanisms within a population are influenced by external interventions (Karamitros et al., 2025; Kyrimi et al., 2025). A randomized experiment is a form of manipulation that uses a randomizing device to assign values to a variable, such as the implementation of a certain diet, rather than relying on naturally occurring mechanisms that dictate dietary choices. In nonexperimental contexts, biologists collect data on gene activation levels in functioning systems to elucidate gene interdependencies and forecast the consequences of manipulating gene expression (Roy, 2025; Wang et al., 2025; Ye et al., 2025). Concurrently, epidemiologists compile data on dietary practices and life expectancy among the general population to identify dietary determinants of longevity and anticipate the outcomes of recommending dietary modifications. Causal inference is characterized by the pursuit of answers on the mechanisms via which variables assume values, or the prediction of a variable’s value following the manipulation of another variable (Keogh and Van Geloven, 2024; Wu et al., 2024a). When only observational (nonexperimental) data are accessible, forecasting the effects of manipulations generally entails sampling from one distribution (of the unmanipulated population) and inferring the values of a variable in a population characterized by a different distribution (of the manipulated population) (Gobonamang and Mpoeleng, 2024; Quillien and Lucas, 2024; Cabañas et al., 2025). A multitude of fundamental issues and assumptions persist consistently across several areas. Moreover, despite certain superficial resemblances between traditional supervised machine learning issues and causal inference, such as the utilization of model search and feature selection, overlapping model types, and the applicability of specific model scores to both domains, these similarities may obscure significant distinctions between the two problem types (Cho et al., 2024; Dahabreh and Bibbins-Domingo, 2024).

A significant share of biomedical and pharmaceutical studies depends on correlation-based machine learning models to analyse experimental and formulation data. While such methods are suitable for prediction and screening, they naturally have limited capability when the main goal is to provide a basis for interventions or to reveal the mechanisms behind them. In the field of formulation science, relationships between formulation variables and performance metrics, such as %EE, do not automatically imply a causal impact. Hence, decisions based solely on correlations might not hold when the parameters are manipulated experimentally. These drawbacks of correlation-based analyses, in turn, partially explain the ongoing trend toward adopting causal inference frameworks. Such frameworks separate the effects of interventions from the associations observed in the data explicitly (Anagnostopoulos et al., 2025). Focusing on the mechanisms that generate the data rather than merely chasing predictive accuracy, causal inference has a sound theoretical justification for addressing counterfactual questions at the heart of rational formulation design. In this new way of looking at problems, determining the value of a causal effect has become a separate task apart from prediction or identifying the most important features. Typical machine learning models, which pinpoint variables most helpful for predictive performance, are very different from causal estimators aimed at measuring the change in an outcome that would result from a hypothetical intervention. The difference is even more significant in biomedical situations characterized by nonlinearities and individual differences in treatment effects.

Tree-based estimators, such as causal forests, are nowadays widely used to estimate conditional average treatment effects from observational data because they easily handle interactions and heterogeneity without relying on strong parametric assumptions (Shiba and Inoue, 2024). Moreover, these approaches emphasize the size and sign of the effect rather than the model’s predictive capabilities, which makes them perfectly aligned with the type of analysis geared towards interventions (Abécassis et al., 2025). Alongside the significant focus on effect estimation, learning causal structures has also been an important area in causal machine learning. Methods for learning causal structures infer DAGs that indicate causal relations between the variables and thus provide an interpretable mechanism of how the system works. A range of methods, such as constraint-based, score-based, and functional causal models, has been developed, each based on different underlying assumptions. Recent studies have demonstrated the applicability of causal discovery for biomedical purposes, e.g., disease risk and clinical decision support, even with small sample sizes and mixed data types (Wu et al., 2024b; Zhao and Jia, 2025). However, most applications operate either under the assumption of a known causal structure or rely on a single discovery method, thereby neglecting structural uncertainty. Causal inference has been widely used across areas such as clinical outcomes, genomics, and drug safety analysis (Mesinovic et al., 2025), (Deng et al., 2025), and recent studies have focused on integrating these with sophisticated machine learning architectures for healthcare counterfactual reasoning (Deng et al., 2025; Mesinovic et al., 2025). However, formulation-level causal analysis (particularly application to mechanisms of encapsulation efficiency) is yet to be developed. Most formulation research has focused primarily on predictive modeling or empirical optimization, with very little attention to causal structure learning, causal effect estimation, or counterfactual simulation. This gap has inspired a proposal for a combined causal discovery and causal effect estimation framework specifically designed for pharmaceutical formulation science that would provide both interpretable causal mechanisms and intervention-ready insights (Bazgir et al., 2025).

Many studies in pharmaceutical formulation rely on correlation-based machine learning models to relate formulation variables to performance metrics such as encapsulation efficiency (%EE). While these approaches are effective for prediction, they provide limited insight into how changes in variables may influence outcomes under intervention. In formulation systems, statistical associations do not necessarily reflect causal relationships, and decisions based solely on predictive importance may not translate into experimental outcomes. Causal inference offers an alternative framework by explicitly modeling the mechanisms through which variables influence one another. Rather than focusing on prediction accuracy, causal methods aim to estimate the effect of interventions and to support counterfactual reasoning. Despite growing applications in biomedical research, their use in formulation science particularly for understanding encapsulation mechanisms remains limited. This study applies a multi-paradigm causal discovery and effect estimation framework to investigate the structural relationships governing %EE in niosomal systems, with the aim of supporting intervention-oriented reasoning.

## 2 Dataset & preprocessing methods

### 2.1 Dataset purpose and construction

The data collected for this research represent formulation-level observations and constitute a fully deterministic data set that, if used in a causal structure learning framework, will accurately identify the underlying causal model. It is not a data set suitable for predictive modeling or empirical optimization. All data points correspond to real experimental formulations reported in peer-reviewed papers on niosomal drug delivery systems, and the ultimate goal was to discover the mechanistic relationships governing %EE. A comprehensive literature search through PubMed and Google Scholar was undertaken. PubMed yielded 49 records, and Google Scholar yielded 114, based on the set of keywords relating to niosomes, Span 60, and TFH. The selection of articles was conducted in accordance with the PRISMA guidelines to ensure a transparent, repeatable process. The dataset consists of 116 samples extracted from 17 (Shahiwala et al., 2023; Arafa and Ayoub, 2017; Nasr et al., 2008; Samifa et al., 2015; Sankhyan and Pawar, 2013; Bhattacharya, 2020; Akbarzadeh et al., 2020; Khalifa and Abdul Rasool, 2017; Moghddam et al., 2016; Ruckmani and Sankar, 2010; Kamboj et al., 2014; Jadon et al., 2009; Tamizharasi et al., 2009; Ramadan et al., 2020; Teaima et al., 2020; Shirsand et al., 2012; Qumbar et al., 2017; Das and Palei, 2011) independent studies and should be interpreted within the limitations of literature-derived observational data. Variability in experimental protocols, measurement techniques, and reporting standards across studies may introduce uncontrolled heterogeneity and potential bias. While restricting the dataset to thin-film hydration formulations improves comparability, it does not eliminate all sources of variability. Therefore, the dataset is not intended to support statistically robust predictive modeling, but rather to enable structured, assumption-aware causal exploration. The selection of formulation and physicochemical variables was based on three criteria: (i) consistent reporting across the selected studies, (ii) established relevance to niosomal formulation behavior, and (iii) availability without ambiguity in the extracted data. No outcome-driven feature selection or data-dependent filtering was applied. This approach ensures that the analysis reflects commonly reported formulation characteristics rather than variables chosen based on their apparent relationship with %EE.

The response variable selected for this study is encapsulation efficiency (%EE), which is the most consistently reported metric across the collected literature sources. While alternative metrics such as drug loading (%DL) may provide a more direct representation of the amount of drug entrapped within vesicular systems, their reporting is inconsistent and often based on differing normalization schemes (e.g., relative to lipid mass or total formulation mass). The inclusion of %DL would therefore substantially reduce the dataset size and introduce additional heterogeneity. Consequently, %EE is employed in this study as a standardized, literature-compatible response variable that enables comparative causal analysis across independently reported formulations.

### 2.2 Controlled heterogeneity and observational nature

To enhance the study’s causal interpretability, the authors chose to focus only on formulations prepared by the thin-film hydration method; in other words, the dataset was intentionally limited to those samples prepared by the thin-film hydration method. Therefore, the authors deliberately imposed a restriction on the dataset: only formulations obtained from the thin-film hydration method were included to increase causal interpretability. This limitation reduces heterogeneity in the effects of different processes and the confounding introduced by using different preparation techniques simultaneously, thereby enabling more interpretable causal structure learning. Since the variables were not changed and the authors performed no experiments, the dataset reflects variations in naturally occurring, independently reported formulations. Hence, the authors did not intervene by manipulating any variables, and they did not conduct any experiments. Thus, the dataset represents naturally occurring variation across independently reported formulations, making it observational. The dataset captures eleven formulation parameters that are both commonly reported and chemically pertinent to niosomal drug delivery systems: molecular weight (g/mol), Log P, aqueous solubility (mg/mL), pKa, hydration medium volume (mL), solvent volume (mL), HLB, drug-to-lipid ratio, cholesterol-to-surfactant molar ratio, hydration time, and hydration temperature. The outcome variable is %EE. The selection of variables was based on the relevance and consistency of reporting, without using outcome-driven feature selection.

### 2.3 Preprocessing strategy and interpretation framing

Preprocessing was deliberately kept minimal in order to maintain the conditional dependencies necessary for causal discovery. At the data extraction stage, samples with missing or ambiguous values were removed, and no imputation was performed. No feature engineering, dimensionality reduction, or outcome-dependent transformations were applied. Continuous variables were standardized only to ensure numerical stability, without altering the relational structure. Given that the data are literature-derived and observational, all analyses that follow are conducted under standard causal assumptions. The resulting findings are seen as hypothesis-generating and are meant to facilitate counterfactual reasoning and decision-making in formulation, rather than to offer confirmatory causal claims.

It should be noted that %EE represents a mass-balance-derived metric, typically calculated from the difference between the total drug added and the amount detected in the supernatant. As such, it does not directly quantify the exact amount of drug physically entrapped within vesicles. In this work, %EE is interpreted as an operational indicator of encapsulation performance, suitable for comparative and causal structure analysis under consistent reporting conditions.

## 3 Methodology

### 3.1 Causal objective and analytical perspective

The research methodology of this paper is deliberately designed to identify causal mechanisms underlying %EE in niosomal formulations, rather than to measure associations or predict performance. The study is based on formulation observation data extracted from the published literature. It is aimed at intervention-relevant reasoning, gaining mechanistically interpretable insights, and conducting counterfactual investigation. Therefore, all findings are considered hypothesis-generating rather than causal-certainty-confirmatory evidence. The entire workflow is a natural causal reasoning pipeline, i.e., causal structure discovery, structure-informed causal effect estimation, and counterfactual analysis.

### 3.2 Causal assumptions

All the steps of our methodology have been implemented according to a set of standard assumptions commonly used in observational causal discovery. Among these are: (i) Acyclicity, which means that the causal system can be represented by a directed acyclic graph (DAG); (ii) no feedback loops between the formulation variables; (iii) causal mechanisms being stable across different formulations; and (iv) causal sufficiency of the observed set, while still acknowledging that unmeasured confounding cannot be ruled out completely. The assumptions are considered as interpretive conditions rather than preconditions, and the causal statements are always explicitly conditioned on them.

### 3.3 Multi-paradigm causal structure discovery

To derive likely causal relationships between formulation parameters and %EE, a multi-paradigm causal discovery strategy was used. It is an approach that benefits from synergistic assumptions to assess the consistency of the inferred relations across different causal frameworks (Niu et al., 2024).

#### 3.3.1 Constraint-based discovery: PC algorithm

The PC algorithm infers causal structure by testing conditional independencies implied by the Markov property of a DAG. For variables $X_{i}$ and $X_{j}$ , the algorithm evaluates relations of the form Equation 1:

$$X_{i}⊥⊥X_{j}|S,S\subseteq X\setminus \left\{X_{i},X_{j}\right\}$$ (1)

Conditionally independent edges are removed, and the rest of the edges are oriented according to logical orientation rules that are consistent with the acyclicity and collider structures. The result is a Markov equivalence class of DAGs, which is represented as a partially directed acyclic graph (Chang et al., 2024).

#### 3.3.2 Score-based discovery: greedy equivalence search (GES)

GES searches over equivalence classes of DAGs by optimizing a penalized likelihood score. For a candidate graph G , the score is defined as Equation 2:

$$\text{Score}\left(G|X\right)=\log p\left(X|G\right)-\lambda \cdot \dim\left(G\right)$$ (2)

Where $\log p\left(X|G\right)$ denotes the data likelihood given the graph structure, $\dim\left(G\right)$ represents model complexity, and λ is a regularization parameter. The algorithm proceeds greedily, adding and deleting edges, to identify structures that balance explanatory adequacy and parsimony (Nazaret and Blei, 2025).

#### 3.3.3 Functional causal modeling: linear non-gaussian acyclic model (LiNGAM)

LiNGAM assumes a linear, non-Gaussian, acyclic data-generating process, expressed in Equation 3:

X=BX+ε (3)

Where B is a strictly lower triangular coefficient matrix consistent with a DAG, and ε is a vector of mutually independent, non-Gaussian noise terms. Under these assumptions, the causal ordering and directed edges are identifiable. LiNGAM yields entirely directed candidate causal graphs that satisfy the assumptions (Yang et al., 2024).

### 3.4 Structural consistency and causal interpretation

Instead of emphasizing a single accurate causal model, the authors examine the estimated graphs for structural consistency, cross-method agreement, and domain plausibility. The causal edges in PC, GES, and LiNGAM are considered candidate mechanistic dependencies. In contrast, the edges that are unstable or specific to the methods are being handled with caution. Doing this kind of triangulation makes them less sensitive to the assumptions of a single algorithm and to the problem of small samples.

### 3.5 Structure-informed causal effect estimation

The causal structure learning methods investigated in this study, PC, GES, and LiNGAM, do not use gradient-based optimization for their operation. The PC algorithm is based on conditional independence testing; GES searches graph equivalence classes via discrete score-based search; and LiNGAM determines the causal order via closed-form estimation under non-Gaussianity assumptions. Hence, gradient-based optimization is not part of the structure learning stage. The sole element of internal optimization is the causal forest for effect estimation, which uses tree-based splitting criteria instead of gradient descent. Causal effect estimation is done after and conditional upon causal structure learning. To measure the size and variation of intervention effects on %EE, a causal forest model is used. Let Y denote %EE, T a formulation parameter treated as an intervention variable, and X the remaining covariates. The conditional average treatment effect (CATE) is defined in Equation 4:

$$\tau \left(x\right)=E\left[Y|\text{do}\left(T=1\right),X=x\right]-E\left[Y|\text{do}\left(T=0\right),X=x\right]$$ (4)

Causal forests estimate $\tau \left(x\right)$ by aggregating treatment effect estimates across an ensemble of decision trees, each optimized through internal, non-gradient-based splitting criteria. This approach enables flexible modeling of nonlinearities and heterogeneous formulation responses without reliance on parametric functional forms.

### 3.6 Counterfactual reasoning and intervention scenarios

Counterfactual analyses are performed to investigate hypothetical formulation interventions, such as incremental changes in hydration temperature. These analyses use the estimated causal effects to measure the differences in the following form of Equation 5:

$$E\left[Y_{T=t^{\prime }}|X=x\right]-E\left[Y_{T=t}|X=x\right]$$ (5)

where $Y_{T=t}$ Denotes the potential outcome under intervention T=t . Counterfactual results are interpreted as reasoning tools to support intervention planning and experimental prioritization, rather than as predictive guarantees.

### 3.7 Methodological scope and limitations

All inferences derived from this model depend on the stated assumptions and the data’s observational nature. The suggested method is not a substitute for controlled experiments. Instead, it provides a causally grounded and interpretable complement that can be used to design the formulation and plan the future experiment. The obtained results thus ought to be regarded as mechanistically informative and hypothesis-generating, and can later be tested empirically. The results of this study should be interpreted in light of several limitations. The relatively small sample size and the use of literature-derived observational data introduce uncertainty related to sampling variability, measurement inconsistency, and potential hidden confounding. In addition, causal discovery algorithms may exhibit sensitivity to data sparsity and noise. Consequently, the inferred causal structures and effect estimates are not intended as definitive representations of the underlying system, but rather as structured hypotheses that require experimental validation. The framework is therefore best understood as a tool for guiding reasoning and prioritizing experimental investigation under uncertainty.

## 4 Results and discussion

### 4.1 Causal structure learning from formulation data

**FIGURE 1:**
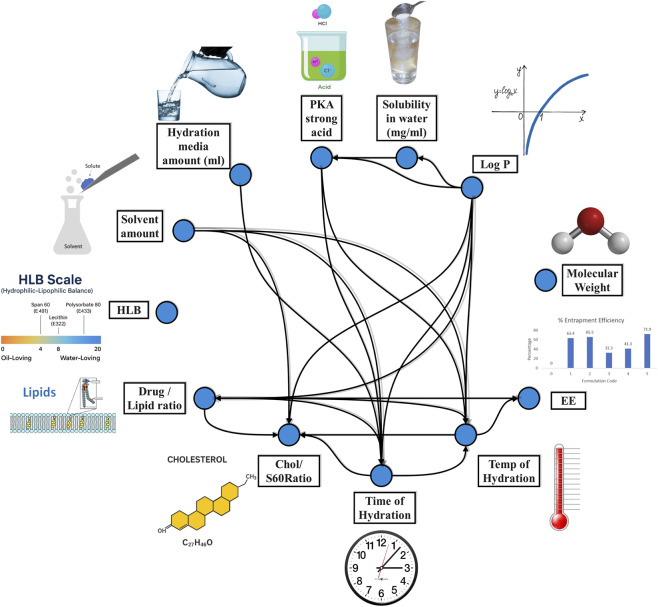
LiNGAM-derived causal graph of formulation parameters affecting encapsulation efficiency (%EE). Directed edges represent inferred causal effects, revealing direct and mediated pathways from formulation, physicochemical, and process variables to %EE and enabling intervention-based interpretation.

Figure 1 shows the LiNGAM-derived causal DAG hypothesizing causal relations between formulation parameters, physicochemical drug properties, process conditions, and %EE. Assuming the LiNGAM model assumptions are correct, the graph provides a single causal explanation consistent with the data rather than a definitive or verified causal structure, and is intended to support intervention-oriented interpretations. Using directed and asymmetric arrows; the arrows actually encode hypothesized cause-and-effect directions. This allows to show how changes in controllable variables can propagate through the system. In this context, %EE is a node in the downstream at which different causal paths from formulation composition, processing variables, and intrinsic drug characteristics converge, thereby mirroring the multilevel nature of niosomal encapsulation. Among process variables, hydration temperature and hydration time have a direct causal effect on %EE, consistent with non-mediated effects (i.e., direct influences) and with the known sensitivity of vesicle formation to thermal and temporal conditions.

On the other hand, several formulation-related variables exert their influence through different paths. For example, the cholesterol-to-surfactant molar ratio indirectly affects %EE via the drug-to-lipid ratio, indicating that lipid composition primarily determines the extent of encapsulation by limiting loading capacity rather than exerting a direct effect. Physicochemical drug properties are depicted upstream in the DAG. Their connections reveal intrinsic dependencies, with Log P as the most important mediator linking solubility-related properties to %EE, which aligns with hydrophobic partitioning behavior. One should see these relations as model-dependent causal hypotheses based on observational data; they are meant to help with intervention reasoning rather than to establish causal mechanisms.

**FIGURE 2:**
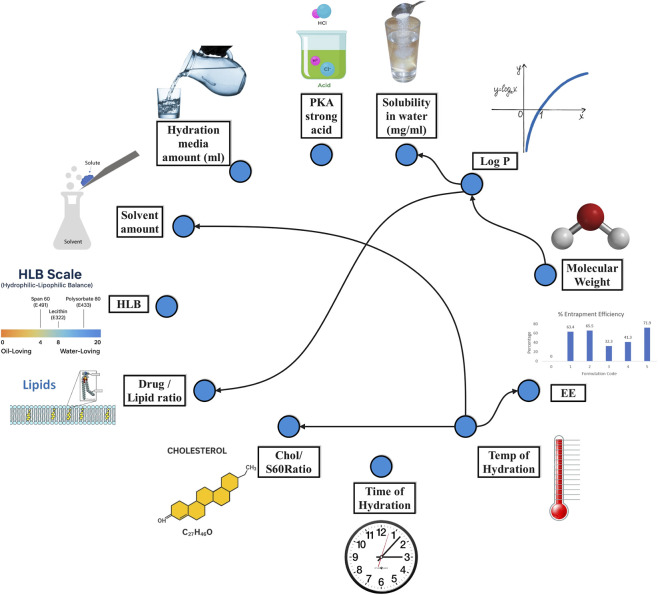
PC-learned causal graph showing directed relationships between formulation parameters, physicochemical properties, and encapsulation efficiency (EE).

Figure 2 displays the causal DAG generated by the PC algorithm, which depicts a model-dependent causal hypothesis based on its conditional-independence and faithfulness assumptions. Since the PC framework is a constraint-based method, it finds relationships in the data that are consistent with the observed ones while remaining deliberately conservative; hence, the resulting graph should not be taken as an actual causal structure. The PC was used to infer this DAG, which is presented here as an alternative to the LiNGAM-derived graph in Figure 1, thus enabling cross-comparison of the two causal discovery paradigms and providing a clue to the robustness of the recurring structural elements. The graph displays a sparser topology resulting from the systematic elimination of edges that were not supported by the conditional-independence testing. Such sparsity is regarded as statistical caution rather than proof of causal minimality or correctness. Most of the directionality in the PC graph has been resolved, which is the typical expected outcome of this framework; some relationships are indirectly constrained rather than fully oriented. Therefore, interpretation primarily focuses on the structure of the overall system rather than making edge-by-edge claims. In this network, EE, as in the previous instance, is a downstream convergence point, with the effect drawn only from a limited subset of formulation and process variables. Similar patterns were observed in the results obtained by the two methods. Hydration temperature maintains its direct link to EE; thus, the results confirm a significant influence of the process conditions, whereas the drug-to-lipid and cholesterol-to-surfactant ratios, as formulation variables, primarily act on EE through indirect pathways. The physicochemical properties are again at the top of the hierarchy, with Log P serving as the intermediate that connects molecular characteristics with formulation-level ones. These are just working hypotheses derived from the data and are to be used for intervention-driven thinking rather than for confirming causal mechanisms.

Table 1 displays a minimal set of inferred directed causality links from the PC algorithm. In fact, the table includes only those links that, after testing for conditional independence, still have a reasonable direction of causality. These links are thus understood as model- and assumption-dependent causal hypotheses, that is, assumptions susceptible to change in light of new evidence, not confirmed mechanisms. They are aimed at supporting intervention-oriented reasoning while also providing a way to avoid overinterpretation of observational data. Directed relationships offer a glimpse into the hierarchical arrangement of variables in the inferred causal structure. Drug physicochemical properties are top-level components characterized by molecular weight pointing toward Log P, and, after that, Log P pointing toward aqueous solubility and the drug-to-lipid ratio. The edges between these chemical properties mainly indicate that the intrinsic physicochemical characteristics are interdependent. Hence, it is clear that molecular features dictate some relationships and, therefore, they cannot be changed solely by formulation design. This is an important point to keep in mind, as it helps us avoid misconceptions about variables whose values are fixed by the structure’s nature. The process variables are shown as downstream, but they still have a significant structural effect. Hydration temperature, as the cause of changes in solvent amount, cholesterol-to-surfactant ratio, and EE, is indicated by the directed edges emanating from temperature to these variables. Hence, temperature is a variable that impacts EE, both directly and indirectly through the formulation composition. The direct influence on EE, unmediated by any mediating paths, is suggested by the direct edge from the process to EE; on the other hand, the additional directed edges represent mediating paths.

**TABLE 1:** Directed causal edges inferred by the PC algorithm.

Source	Target	Attributes
Molecular weight	Log P	Relationship-directed
Log P	Solubility in water (mg/mL)	Relationship-directed
Log P	Drug/Lipid ratio	Relationship-directed
Temp of hydration	Solvent amount	Relationship-directed
Temp of hydration	Chol/S60Ratio	Relationship-directed
Temp of hydration	EE	Relationship-directed

The LiNGAM model was employed to infer the directed causal relationships, and the corresponding weights are presented in Table 2; all results depend on the assumptions of linearity, non-Gaussian noise, and causal sufficiency. The numbers shown are model-dependent causal weights that reflect only the relative sign and magnitude of causal influence within the inferred DAG; they should not be treated as empirically verified effect sizes or parameters that can be transported. Besides the graphical framework, the table offers drug physicochemical properties that occupy higher levels in the formulation system hierarchy. Log P turns out to be a structurally influential variable that bridges the intrinsic molecular features, formulation composition, and process conditions. The negative causal weight from Log P to aqueous solubility agrees with the well-known hydrophobic-hydrophilic trade-offs. At the same time, its directed relationships with formulation ratios and processing variables indicate that molecular hydrophobicity imposes a limitation on production regimes compatible with the drug rather than being production-driven. Further down, drug and production variables are shown as intervention-relevant tools. Drug hydration temperature and drug-to-lipid ratio have a direct, positive causal relationship with EE; thus, they are also correlated with multi-step-mediated pathways in which hydration time and cholesterol composition play a role. These indirect routes reveal that EE results from the clouding of causal mechanisms interacting with one another.

**TABLE 2:** Causal relationships and edge weights inferred by the LiNGAM model.

Source	Target	Attributes (weight)
Log P	Solubility in water (mg/mL)	*−7.076*
Log P	PKA strong acid	*3.343*
Log P	Drug/Lipid ratio	*−0.519*
Log P	Chol/S60Ratio	*0.366*
Log P	Time of hydration	*0.425*
Log P	Temp of hydration	*1.068*
Solubility in water (mg/mL)	PKA strong acid	*0.346*
Solubility in water (mg/mL)	Time of hydration	*0.092*
Solubility in water (mg/mL)	Temp of hydration	*0.109*
PKA strong acid	Time of hydration	*−0.111*
PKA strong acid	Temp of hydration	*−0.183*
Hydration media amount (mL)	Time of hydration	*−0.094*
Solvent amount	Chol/S60Ratio	*0.239*
Solvent amount	Time of hydration	*0.289*
Solvent amount	Temp of hydration	*0.515*
Drug/Lipid ratio	Chol/S60Ratio	*−0.132*
Drug/Lipid ratio	EE	*5.548*
Time of hydration	Drug/Lipid ratio	*0.293*
Time of hydration	Chol/S60Ratio	*−0.362*
Time of hydration	Temp of hydration	*−0.441*
Temp of hydration	Drug/Lipid ratio	*0.174*
Temp of hydration	Chol/S60Ratio	*−0.611*
Temp of hydration	EE	*6.994*

### 4.2 Cross-method agreement and consensus causal graph

**FIGURE 3:**
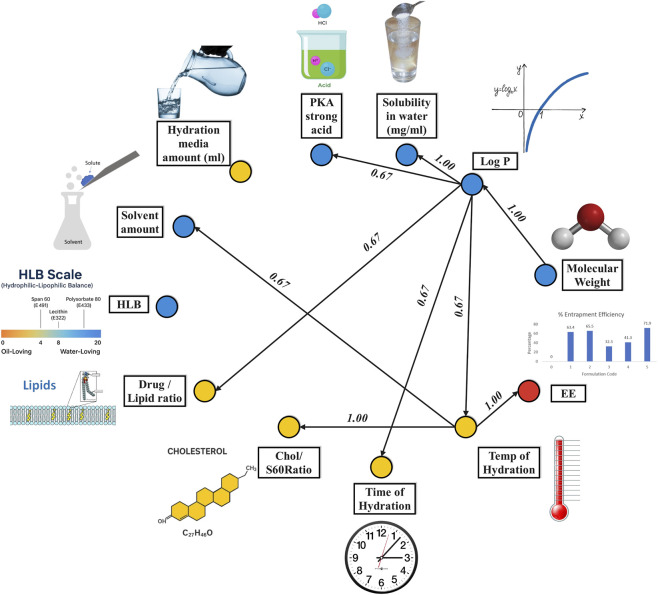
Consensus causal DAG for %EE obtained from PC, GES, and LiNGAM. Edges represent robust causal relations with normalized strengths, highlighting the key formulation and process variables that directly govern encapsulation efficiency.

Figure 3 depicts an assumption-aware consensus causal DAG for %EE, resulting from integrating the causal structures independently inferred using the PC, GES, and LiNGAM algorithms. By integrating constraint-based, score-based, and functional causal discovery paradigms, this consensus graph selects causal relations that are structurally stable across very different sets of assumptions; it therefore does not claim to be a final or accurate causal model. Hence, method agreement is seen as robustness across different algorithms, rather than a procedure for causal validation. Edges in the consensus DAG correspond to method-consistent causal hypotheses, with normalized strengths expressing the number of and the internal consistency of the directional agreement among the algorithms used, rather than the magnitude or effect size of the causal. Thus, this rereading retains the emphasis on the stability of directionality and explicitly avoids the variable ranking or the optimization-based interpretation. As the main %EE node in a causal structure, it is influenced only by a minimal set of formulation and process variables. More specifically, hydration temperature and the drug-to-lipid ratio are directly related to %EE, depending on the methods used. They may be considered stable intervention candidates in the inferred causal system; however, this does not imply they have the greatest impact or are optimal. Physicochemical drug properties, in particular Log P, are first-level influences and thus form a link between the molecular characteristics of the drug and formulations, as well as process conditions; they provide structural constraints. Given that mediated paths remain through variables such as cholesterol ratio and hydration time, it is also indicated that encapsulation efficiency results from the interaction of causal mechanisms rather than isolated effects.

**FIGURE 4:**
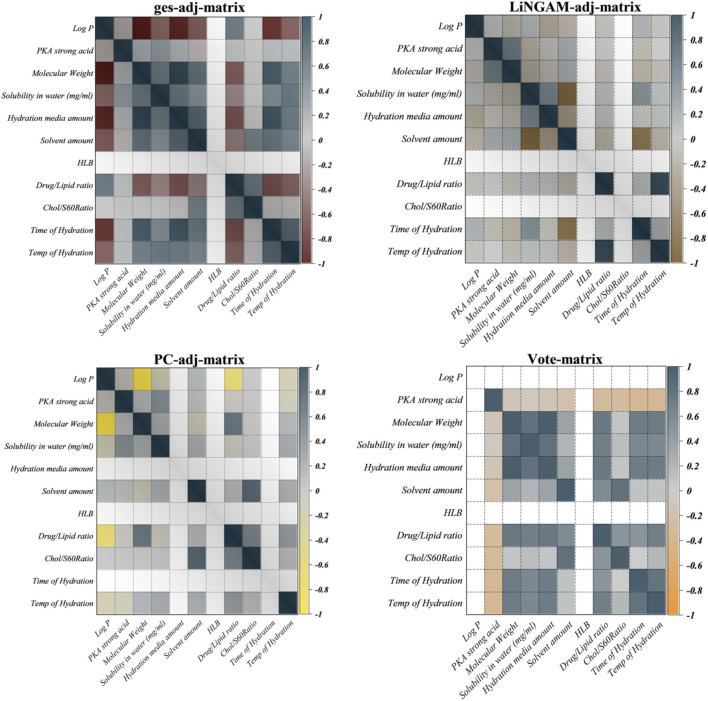
Adjacency matrices inferred by GES, LiNGAM, and PC algorithms, along with the resulting consensus (vote) matrix summarizing agreement and directionality of causal relationships among formulation variables.

Figure 4 shows the adjacency matrices inferred by GES, LiNGAM, and PC without knowledge of each other’s work, along with a vote-based consensus matrix that reflects the overall directional agreement among the methods. This figure primarily serves as a diagnostic tool for assessing the stability of causal findings across different discovery paradigms; it directly considers the extent to which the inferred causal relations vary across different causal discovery algorithms. It does not aim to establish causal validity or confirm causality, but rather to aid in interpreting results based on their robustness. Each adjacency matrix is essentially a pictorial representation of the causal hypotheses put forward by that particular algorithm, and the observed structural variations among them are not only expected but also quite revealing. For instance, the PC algorithm produces a less connected graph, which aligns well with its cautious approach to conditional-independence testing.

In contrast, both GES and LiNGAM tend to generate denser graphs due to score-based optimization and functional modeling assumptions, respectively. These variations ought to be seen as the natural theoretical ramifications of distinct causal assumptions rather than as a methodological disagreement or weakness, thereby highlighting the importance of integrating rather than relying solely on a single model. These matrices, together with a vote-based synthesis, articulate cross-algorithm uncertainty, an aspect that is rarely divulged in formulation-level causal analyses. The vote matrix thus serves as a meta-level causal summary, identifying the causal directions that recur across different algorithms despite their varying assumptions. It is crucial to note that entries other than zero in the vote matrix merely indicate how often and how consistently a given direction is agreed upon, rather than the causal effect size, strength, importance, or confidence. Comparing from this robustness perspective not only guides the choice of edges left in the consensus DAG but also helps rationally prioritize those edges most suitable for counterfactual and intervention-oriented reasoning, all the while being firmly grounded in observation and literature-derived data.

**FIGURE 5:**
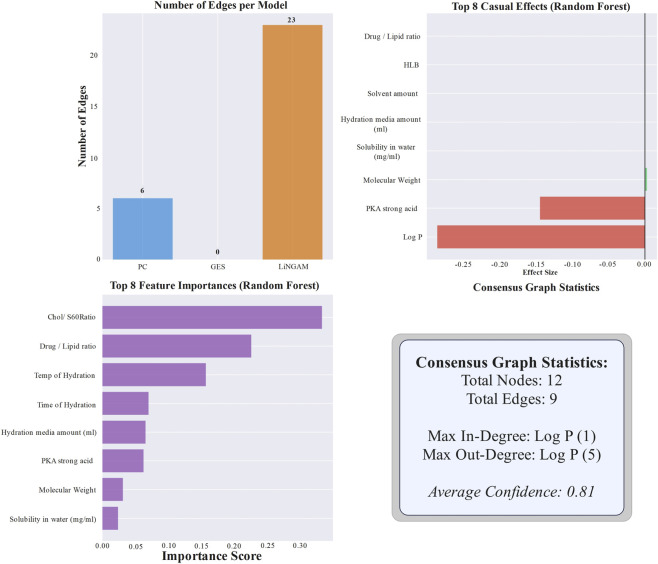
Comparison of causal structure complexity, estimated causal effects, feature importances, and summary statistics of the consensus causal graph derived from formulation data.

Figure 5 offers a triangulation of causal structure, algorithmic sensitivity, and predictive relevance for formulation variables from several angles, mainly for the sake of contextual interpretation rather than validation. It combines various sources of complementary information from causal discovery results, model-conditional causal effect estimations, and RF feature importances in order to uncover areas of both agreement and disagreement. The upper-left panel illustrates a comparison of structural complexity among different causal discovery algorithms. As the panel shows, PC results in a very sparse graph, LiNGAM generates a quite dense network, and GES is somewhere in the middle. These variations come from assumption-driven differences and should not be considered mistakes.

What is more, they encourage the use of a consensus graph to reveal structurally stable relations. The upper-right panel depicts causal effect estimates from RF, which indicate the directions of associations conditional on model assumptions. The latter are therefore helpful in providing directional context for hypothesis generation. However, they ought not to be misconstrued as verified sizes or as proof of one factor having a more causal influence than another. The bottom-left panel displays RF feature importances, which focus on predictive rather than causal contributions. Cholesterol ratio, drug-to-lipid ratio, and hydration parameters are among the variables that partially align with recurrent causal edges, thus giving an example of contextual consistency without necessarily indicating that they are directly controllable. At last, consensus graph statistics are features that describe system-level properties, such as node connectivity, edge counts, and network centralization, among others, thereby shedding light on the hierarchical interactions among molecular, formulation, and process variables.

### 4.3 Quantitative causal effect estimation

**FIGURE 6:**
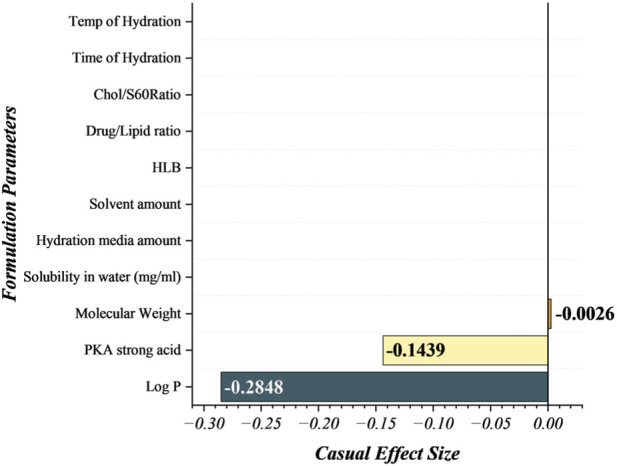
Estimated causal effects of formulation and physicochemical parameters on encapsulation efficiency (%EE).

Figure 6 is a summary of the estimated direct causal effects of formulation and physicochemical variables on %EE obtained by a Causal Forest model conditioned on the learned DAG. The reported effect sizes are the average intervention-based changes in %EE, explicitly adjusted for confounding and mediation. They should therefore be interpreted as causal associations rather than correlations. Of all the variables, Log P has the most substantial direct adverse causal effect on %EE, with an effect size of more than 0.285; i.e., an increase in drug lipophilicity causally decreases %EE as a result of the intervention. After accounting for the causal structure, this impact persists, suggesting that the primary way lipophilicity affects the system is not through formulation variables. The pKa of strong acidic groups also shows a moderate negative direct causal effect of approximately 0.144, indicating that ionization behavior is a causal mechanism in drug bilayer interactions during encapsulation.

On the other hand, the molecular weight shows a tiny direct causal effect (close to 0.003); thus, its apparent importance in predictive models is primarily due to a non-direct effect. In general, these process-related formulation parameters, such as hydration temperature, hydration time, solvent amount, hydration medium volume, surfactant composition, HLB, drug/lipid ratio, and cholesterol-to-surfactant ratio, yield effect size estimates close to zero, with the confidence intervals covering the null effect. Under the experimental conditions used in the literature-derived dataset, these parameters do not directly and strongly affect %EE; however, their indirect or interaction effects cannot be completely ruled out. Most importantly, these results surpass traditional correlational analyses and provide intervention-based causal estimates, thereby allowing rational counterfactual reasoning.

### 4.4 Predictive feature contributions (non-causal benchmark)

**FIGURE 7:**
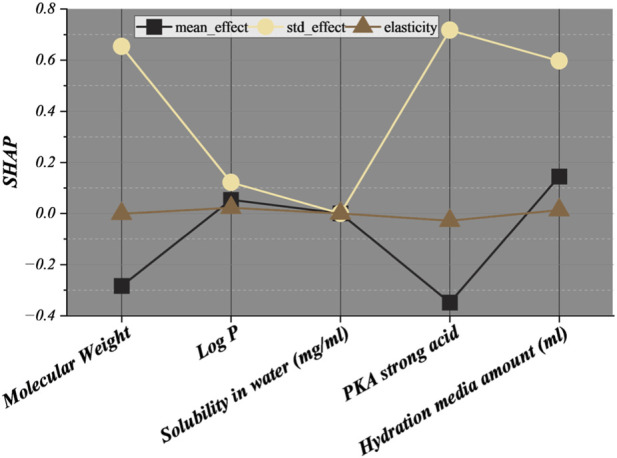
Comparison of mean SHAP values, variability, and elasticity for key physicochemical and formulation variables influencing encapsulation efficiency (%EE).

Figure 7 illustrates the quantitative explanation of the prediction of drug %EE by displaying SHAP values and elasticity metrics, thereby showing the difference between model-based attribution and intervention-based causal inference. The mean SHAP values represent the average predictive contribution of each variable to the trained model; their spread indicates formulation-dependent heterogeneity, and elasticity measures the local, relative sensitivity of predictions to changes in inputs. These metrics essentially characterize the model behavior in terms of association and should not be taken as indications of causal influence. Among the three top features, the pKa of strong acidic groups and the amount of hydration media show mean SHAP values of high magnitude, indicating that they play a major role in predicting the model’s outcome. In fact, pKa exhibits a substantial negative mean SHAP value and a high standard deviation, indicating strong context-dependent predictive behavior across the formulations. Molecular weight is another feature that negatively impacts the mean SHAP value but with greater variability; however, it has no direct, significant causal effect in Figure 7, thus illustrating that a feature can have a certain level of predictive relevance while being almost causally irrelevant. Log P, on the other hand, has a small yet relatively steady contribution from SHAP. In contrast, aqueous solubility shows SHAP values that are essentially centered around zero, indicating very little or almost no predictive power. Elasticity metrics remain close to zero across all changes, indicating that the local sensitivities of the prediction %EE are nonlinear and asymmetric.

### 4.5 Causal interpretation of results

Interpretatively, the study helps explain why a causal analytical framework fits very well with formulation datasets derived from diverse literature sources, where, on the one hand, just predictive accuracy is not expected to lead to feasible interventions. On the other hand, it certainly means meaningful interventions. By determining causal structure, the analysis shifts the focus from merely identifying statistical associations to considering how changing formulation and process variables may impact the system under the assumptions of interventions, while keeping the degree of uncertainty about the validity of causal claims at a normal level. Around the node, the %EE is the effect in all the different causal discovery models. Physicochemical properties of the drug are located more upstream, and frequently, Log P is the most important variable explaining how drug molecular properties influence formulation composition and processing.

Furthermore, this attribute is also confirmed by the causal effect estimation, which points out that direct interventions on Log P would cause the most substantial decrease in %EE (average intervention effect size≈ 0.285). This does not prove that hydrophobicity is the only factor; on the contrary, it is the one that structurally limits the drug-bilayer partitioning behavior by defining the viable formulation regimes based on the nature of the drug’s hydrophobicity. On the other hand, molecular weight is rarely considered a highly important factor in predictive studies; the direct causal effect of this parameter is very low thus indicating that it is challenging to distinguish between the two and that the criteria of one of them being dominant should be neglected, Some of the process parameters that are discussed in the literature have been revealed be in the vicinity of %EE and thus more accessible for interventions, for instance, hydration temperature is one of them. LiNGAM assigned it a direct positive impact weight (≈6.99), which was also saved in the consensus structure. On the other hand, mediated pathways involving hydration time and formulation ratios show that temperature no doubt affects, in tandem with other factors, and that it does not simply bring about changes in single-component systems. Components of the formulation, such as the drug-to-lipid and cholesterol-to-surfactant ratios, mainly affect %EE via indirect pathways, so they have quite different contextual effects.

A notable difference is between the causal effect estimates and the predictive feature attributions. Some variables that are found to have high predictive relevance in SHAP analyses include pKa and the amount of hydration media, which, after structural adjustment, show only very little direct causal effect. This points to how predictive models can use correlation and interaction patterns that do not necessarily reflect manipulative causal pathways - a significant distinction in formulation science. In general, the combination of causal structure learning and effect estimation provides a different perspective on conventional ML interpretability, and the results can be considered hypothesis-generating when observational data and the assumptions of the underlying causal model are taken into account.

The interpretation of causal effects should also be considered in light of the response variable used. Encapsulation efficiency (%EE), while widely reported, reflects a bulk mass-balance estimate rather than a direct measurement of vesicle-level drug content. Metrics such as drug loading (%DL) may offer a more physically representative characterization of encapsulated drug fraction. However, due to limited and inconsistent reporting of %DL in the literature, such analysis could not be robustly implemented in the present study. Future extensions of this framework could incorporate %DL-based causal analysis, which may provide complementary insights into formulation efficiency from a vesicle-normalized perspective.

### 4.6 Real-world implications

From a practical perspective, this work demonstrates how causal analysis can be used to inform formulation reasoning in situations where the use of predictive models for accuracy does not directly imply the feasibility of the intervention. More than prescribing formulation rules or the best conditions, the causal perspective introduced here gives a structured way to think about different classes of variables and how those relationships could be a source of information for planning under uncertainty. Within this framing, causal models serve as decision-support tools that help organize hypotheses in researchers’ minds rather than as substitutes for experimental validation.

One possible implication is the prioritization of experiments during early-stage formulation development. The consistent positioning of physicochemical drug properties, especially Log P, at structurally upstream positions indicates that these variables may serve as a limit on achievable encapsulation behavior in the analyzed context. Although these properties are frequently highlighted in predictive modeling, their causal location suggests that they are only moderately responsive to downstream process changes. Being aware of these limits can be very useful in making early-stage decisions about experiments likely to be informative and those likely to be unresponsive to interventions.

A related implication concerns the relevance of interventions between formulation and process variables. Different process parameters, e.g., hydration temperature, are, according to the model’s assumptions, more directly related to encapsulation efficiency and can, in principle, be adjusted during formulation development. This closeness at the level of process steps or stages does not necessarily mean that the corresponding feature can be improved. However, it indicates that changes in sheltering agent ratios might be less efficient candidates than specific process parameters for a systematic investigation. Such differentiation may, without prescriptive control, provide avenues for experimental framing.

The results also point to implications for the machine learning workflows in formulation science. The discrepancy between predictive feature importance and causal effect estimates demonstrates the limitations of methods that depend solely on interpretability metrics derived directly from predictive models. Features that increase prediction accuracy might do so because of, e.g., the presence of correlation patterns or interactions that are not convertible into the causality of the source. Thus, adding a causal approach to predictive modeling might help explain a variable’s ability to be either informative for intervention or for explanation.

**FIGURE 8:**
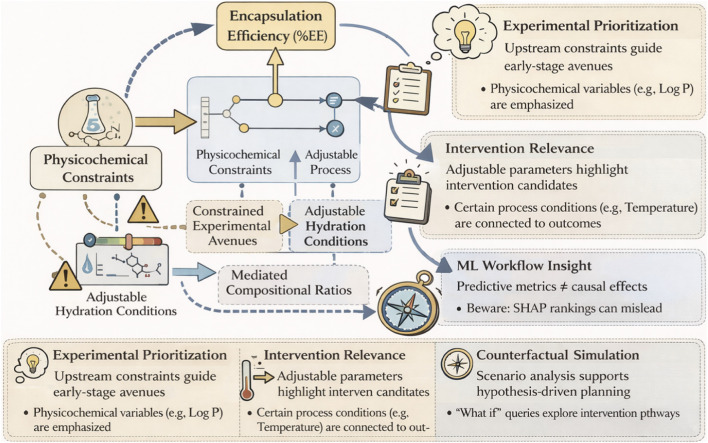
Conceptual overview illustrating how causal structure learning and counterfactual reasoning support formulation decision-making by distinguishing physicochemical constraints, adjustable process variables, and predictive versus intervention-relevant insights for encapsulation efficiency (%EE).

Lastly, causal structure learning and counterfactual reasoning pave the way for explanatory research and thus for capacity building among experimental investigators through scenario analyses. Counterfactual queries here should be understood mainly as “what-if scenarios” scenarios based on the model’s assumptions, rather than as experimental outcomes. These analyses may help researchers design a more targeted experiment and better understand it, whilst at the same time maintaining consistency with the observational nature of the database (Figure 8).

## 5 Conclusion

This research takes a causal, structure-oriented perspective to explain the formulation parameters affecting %EE in niosomal drug delivery systems. The authors argue that relying on predictive accuracy alone hinders intervention-oriented decision-making. The study uses observational, literature-based data for causal discovery and effect estimation, not to prove definitive mechanisms, but to demonstrate how formulation variables are structurally related to each other and how these relationships may serve as examples of causal reasoning under explicit assumptions. One of the main theoretical contributions is that the authors reframe the formulation variables according to their positions in the inferred causal structure. In fact, using various causal discovery paradigms, the authors show that %EE is consistently treated as a downstream outcome influenced by only a few variables, whereas physicochemical drug properties are included at the upstream end. This structural dichotomy implies that some variables serve merely as molecular constraints, while others are potentially amenable to intervention through formulation and process design. Accordingly, the focus is shifted from the search for “important predictors” to variables that can respond significantly to intervention and that, on the contrary, mainly set the boundaries of the formulation space.

Another related point is that this study has some consequences for how one should interpret machine learning results in the context of formulation research. The difference in the composition and the interpretation of the result that has been noticed shows that the features determined as essential by the prediction do not necessarily match the intervention levers. Predictive models are designed to capture the data patterns of association and interaction that can result from mediated or non-manipulable paths, while causal modelling explicitly shows the propagation of ripple changes through the system under the assumptions being modeled. This study first presents causal structure learning and predictive benchmarking as separate entities and demonstrates that combining the two yields a more comprehensive perspective for interpreting the models. The conclusions have to be assumption-aware, since the analyses are based on observational data collected from different sources in the literature and depend on standard causal assumptions, such as causal sufficiency and model-specific conditions. Even though the structural agreement of several algorithms provides structural patterns with a degree of confidence, unmeasured confounding and limited variable ranges cannot be ruled out. Therefore, the inferred causal relationships should be considered as generating new hypotheses rather than confirming them. Taken together, this study presents a framework for analyzing formulation data that prioritizes intervention-oriented reasoning over performance-driven interpretation. Explaining how structural restrictions, indirect effects, and potentially changeable variables can play a role, the causal view elaborated here balances a principled formulation with machine learning methods, while acknowledging the limitations of inference from observational data.

Future work may extend the proposed causal framework to alternative response variables such as drug loading (%DL), particularly as more standardized and comprehensive datasets become available, enabling a more direct assessment of vesicle-level drug incorporation.

## Data Availability

The original contributions presented in the study are included in the article/supplementary material, further inquiries can be directed to the corresponding author.

## Nomenclature

EE: Encapsulation Efficiency
DAG: Directed Acyclic Graph
TFH: Thin-Film Hydration
PRISMA: Preferred Reporting Items for Systematic Reviews and Meta-Analyses
HLB: Hydrophilic–Lipophilic Balance
GES: Greedy Equivalence Search
LiNGAM: Linear Non-Gaussian Acyclic Model
PC: Peter–Clark algorithm
ML: Machine Learning
